# *Mycobacterium tuberculosis* hijacks host TRIM21- and NCOA4-dependent ferritinophagy to enhance intracellular growth

**DOI:** 10.1172/JCI159941

**Published:** 2023-04-17

**Authors:** Youchao Dai, Chuanzhi Zhu, Wei Xiao, Kaisong Huang, Xin Wang, Chenyan Shi, Dachuan Lin, Huihua Zhang, Xiaoqian Liu, Bin Peng, Yi Gao, Cui Hua Liu, Baoxue Ge, Stefan H.E. Kaufmann, Carl G. Feng, Xinchun Chen, Yi Cai

**Affiliations:** 1 Guangdong Provincial Key Laboratory of Regional Immunity and Diseases, Department of Pathogen Biology, Shenzhen University Medical School, Shenzhen, China.; 2 Guangzhou Eighth People’s Hospital, Guangzhou Medical University, Guangzhou, China.; 3 Laboratory of Molecular Biology, Beijing Key Laboratory for Drug Resistance Tuberculosis Research, Beijing Chest Hospital, Capital Medical University, Beijing Tuberculosis and Thoracic Tumor Research Institute, Beijing, China.; 4 Shenzhen Bay Laboratory, Shenzhen, China.; 5 Zhuhai Center for Disease Control and Prevention, Zhuhai, China.; 6 First Affiliated Hospital of Guangzhou University of Chinese Medicine, Guangzhou, China.; 7 Integrated Chinese and Western Medicine Postdoctoral Research Station, Jinan University, Guangzhou, China.; 8 Department of Infectious Disease, Shenzhen People’s Hospital, Second Clinical Medical College of Jinan University, Shenzhen, China.; 9 Guangdong Key Laboratory for Genome Stability & Disease Prevention and Carson International Cancer Center, Marshall Laboratory of Biomedical Engineering, Shenzhen University Medical School, Shenzhen, China.; 10 School of Biomedical Engineering, Health Science Center, Shenzhen University, Shenzhen, China.; 11 CAS Key Laboratory of Pathogenic Microbiology and Immunology, Institute of Microbiology, Center for Biosafety Mega-Science, Chinese Academy of Sciences, Beijing, China.; 12 Shanghai Key Laboratory of Tuberculosis, Shanghai Pulmonary Hospital, Tongji University School of Medicine, Shanghai, China.; 13 Max Planck Institute for Infection Biology, Berlin, Germany.; 14 Max Planck Institute for Multidisciplinary Sciences, Göttingen, Germany.; 15 Hagler Institute for Advanced Study, Texas A&M University, College Station, Texas, USA.; 16 Immunology and Host Defense Group, School of Medical Sciences, Faculty of Medicine and Health, The University of Sydney, Sydney, New South Wales, Australia.

**Keywords:** Immunology, Infectious disease, Bacterial infections, Innate immunity

## Abstract

Ferritin, a key regulator of iron homeostasis in macrophages, has been reported to confer host defenses against *Mycobacterium tuberculosis* (Mtb) infection. Nuclear receptor coactivator 4 (NCOA4) was recently identified as a cargo receptor in ferritin degradation. Here, we show that Mtb infection enhanced NCOA4-mediated ferritin degradation in macrophages, which in turn increased the bioavailability of iron to intracellular Mtb and therefore promoted bacterial growth. Of clinical relevance, the upregulation of FTH1 in macrophages was associated with tuberculosis (TB) disease progression in humans. Mechanistically, Mtb infection enhanced NCOA4-mediated ferritin degradation through p38/AKT1- and TRIM21-mediated proteasomal degradation of HERC2, an E3 ligase of NCOA4. Finally, we confirmed that NCOA4 deficiency in myeloid cells expedites the clearance of Mtb infection in a murine model. Together, our findings revealed a strategy by which Mtb hijacks host ferritin metabolism for its own intracellular survival. Therefore, this represents a potential target for host-directed therapy against tuberculosis.

## Introduction

A variety of mechanisms have evolved in mammalian cells to control intracellular pathogens. For example, preventing intracellular pathogens from accessing iron, an essential nutrient for both host and pathogen, by iron-chelating proteins, such as ferritin and lactoferrin, is an important host defense strategy (1, 2). Ferritin, which comprises 24 polypeptide subunits of the ferritin heavy (FTH1) and light chains (FTL), is the central regulator of iron homeostasis in macrophages. Previous studies have demonstrated that deficiency of the *FTH1* gene in bone marrow or myeloid cells significantly increases susceptibility to infection by pathogens, including *Mycobacterium tuberculosis* (Mtb) (3, 4) and *Salmonella typhimurium* (5).

Besides sequestering iron, ferritin supplies iron to the host and pathogen via ferritin degradation (6, 7). Thus, ferritin metabolism could benefit pathogens, due to its role in the release of iron. Consistent with this, recent studies have revealed that pathogens can hijack host ferritin-stored iron via ferritinophagy for their survival and growth (7, 8). In addition, a high abundance of FTH1 and FTL is found in the cellular rim of tuberculosis (TB) cavitary granulomas (9). Furthermore, ferritin was shown to be upregulated in alveolar macrophages but not in interstitial macrophages, corresponding with high and low intracellular Mtb growth, respectively (10). In line with these findings, we found that the ferritin level in serum was positively correlated with the severity of lung damage and bacillary load in patients with TB (11). Together, these findings suggest that increased ferritin could benefit Mtb survival. However, whether and how host ferritin facilitates Mtb growth remains to be elucidated.

In the present study, we sought to determine the effects of ferritin on intracellular Mtb replication, depending on the availability of free iron. We observed that Mtb induces nuclear receptor coactivator 4–mediated (NCOA4-mediated) ferritin degradation for enhanced iron bioavailability and bacterial growth. Further, we found that ferritinophagy in macrophages is involved in human TB disease progression and revealed mechanisms and signaling pathways underlying the hijacking of host ferritin metabolism by Mtb for its replication. Finally, we used an in vivo mouse model with NCOA4-deficient myeloid cells to investigate their role in resistance to Mtb infection. Our study thus identifies the exploitation of host ferritin metabolism as an Mtb strategy for intracellular growth.

## Results

### Detrimental and beneficial roles of ferritin in intracellular Mtb growth are dependent on iron availability.

**Figure 1:**
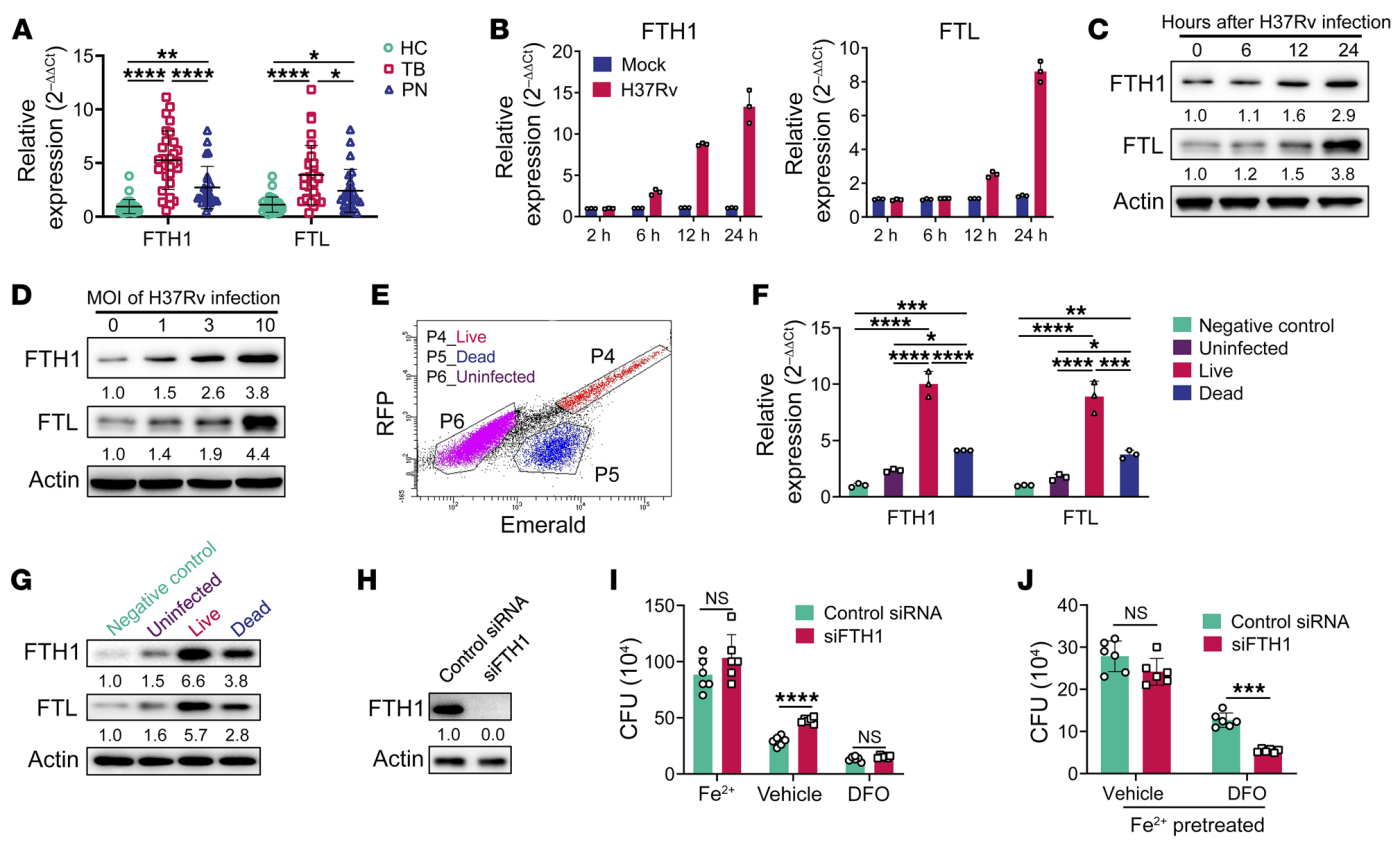
Expression of ferritin is associated with Mtb growth in macrophages. (**A**) Quantitative reverse transcriptase PCR (RT-qPCR) analysis of *FTH1* and *FTL* mRNA abundance in PBMCs from healthy controls (HC, *n* = 35), TB patients (TB, *n* = 30), and non-TB pneumonia patients (PN, *n* = 25). (**B**) RT-qPCR analysis of *FTH1* and *FTL* at different time points (2, 6, 12, and 24 hours) in THP-1–derived macrophages infected or not infected with H37Rv (MOI = 3). (**C** and **D**) Immunoblot analysis of FTH1 and FTL at different time points (0, 6, 12, and 24 hours) in THP-1–derived macrophages after infection with the indicated MOI (0, 1, 3, or 10). (**E**–**G**) THP-1–derived macrophages were infected for 72 hours with Mtb strain H37Ra carrying a dual-color reporter that comprises a constitutively green (Emerald) and a tetracycline-inducible red (TagRFP) fluorescent protein. Tetracycline (500 ng/mL) was added 24 hours before analysis of the live or dead status of the H37Ra strain in macrophages by flow cytometry (**E**). (**F** and **G**) RT-qPCR analysis (**F**) and immunoblot analysis (**G**) of FTH1 and FTL in negative control, uninfected, and harboring live or dead H37Ra macrophages. (**H**) Immunoblot analysis of FTH1 in FTH1-knockdown THP-1–derived macrophages. (**I**) Intracellular H37Rv CFU levels in FTH1-knockdown THP-1–derived macrophages treated with ferrous lactate (20 μM), DFO (25 μM), or vehicle. (**J**) THP-1–derived macrophages with FTH1 knockdown were pretreated with ferrous lactate (20 μM) for 24 hours. Then CFUs of intracellular H37Rv were assessed in the presence of DFO (25 μM) or vehicle. Data in **F**–**J** are representative of 2 or 3 independent experiments. Data are presented as means ± SD; *P < 0.05, **P < 0.01, ***P < 0.001, ****P < 0.0001 by 1-way ANOVA with Tukey’s post hoc test (**A** and **F**) or Student’s 2-tailed unpaired *t* test (**I** and **J**).

Two recent studies have found that FTH1 deficiency in bone marrow or myeloid cells results in significantly increased susceptibility to TB in mice (3, 4); however, we and others found increased serum levels of ferritin in TB patients (11, 12). While increased serum levels of ferritin could be a consequence of Mtb infection, an alternative possibility is that ferritin supports Mtb replication, as suggested by studies of other infection models (7, 8). Consistent with this notion, in the present study both *FTH1* and *FTL* mRNA levels in peripheral blood mononuclear cells (PBMCs) were significantly higher in patients with pulmonary TB than in healthy controls and non-TB pneumonia patients (Figure 1A). Upon infection with Mtb, THP-1 macrophages rapidly upregulated ferritin expression in a time- and dose-dependent manner before increased intracellular Mtb growth (Figure 1, B–D). To further determine the relationship between the expression of host ferritin and intracellular Mtb survival, we compared the expression of ferritin in sorted macrophage populations that harbored live or dead Mtb versus those without bacteria (uninfected), as previously described (13). Macrophages without Mtb exposure were used as a negative control (unexposed) (Figure 1E). In line with the clinical findings outlined above, FTH1 and FTL were significantly increased at both mRNA and protein levels in macrophages harboring live Mtb compared with those harboring dead Mtb or uninfected or unexposed macrophages (Figure 1, F and G). Together, these findings demonstrated that increased ferritin in macrophages correlated with intracellular survival of Mtb.

To further determine whether there is a causal relationship between increased ferritin expression and Mtb growth within macrophages, we used small interfering RNA (siRNA) to knock down the expression of FTH1 and FTL and evaluated the effect on intracellular Mtb growth. The efficacy of knockdown was confirmed by immunoblotting (Figure 1H). There was a compensatory increase in the expression of FTL in FTH1-knockdown macrophages compared with controls (Supplemental Figure 1A; supplemental material available online with this article; https://doi.org/10.1172/JCI159941DS1) (5). In agreement with others (4), we observed that FTH1 knockdown significantly increased the growth of intracellular Mtb, while silencing FTL did not affect Mtb growth in macrophages (Figure 1I and Supplemental Figure 1B). Therefore, for the remainder of the study, we focused on FTH1 to investigate the role of ferritin in controlling bacterial growth.

As iron is vital for Mtb replication in macrophages (14), we further explored the impact of free iron concentration on the role of ferritin in regulating intracellular Mtb growth. To this end, we either chelated iron using deferoxamine (DFO) or supplied iron in the form of ferrous lactate. As expected, the availability of iron promoted bacterial growth, whereas limiting the availability of iron inhibited it (Figure 1I). Notably, the role of FTH1 in restricting intracellular Mtb growth was abrogated by treatment with either DFO or ferrous lactate (Figure 1, I and J). Moreover, the withdrawal of iron from macrophages pretreated with ferrous lactate reversed the supportive effect of FTH1 deficiency on intracellular Mtb growth (Figure 1J). Thus, our data indicate that ferritin is detrimental or beneficial for intracellular Mtb growth depending on the availability of iron.

### NCOA4-mediated ferritin degradation facilitates intracellular Mtb growth.

**Figure 2:**
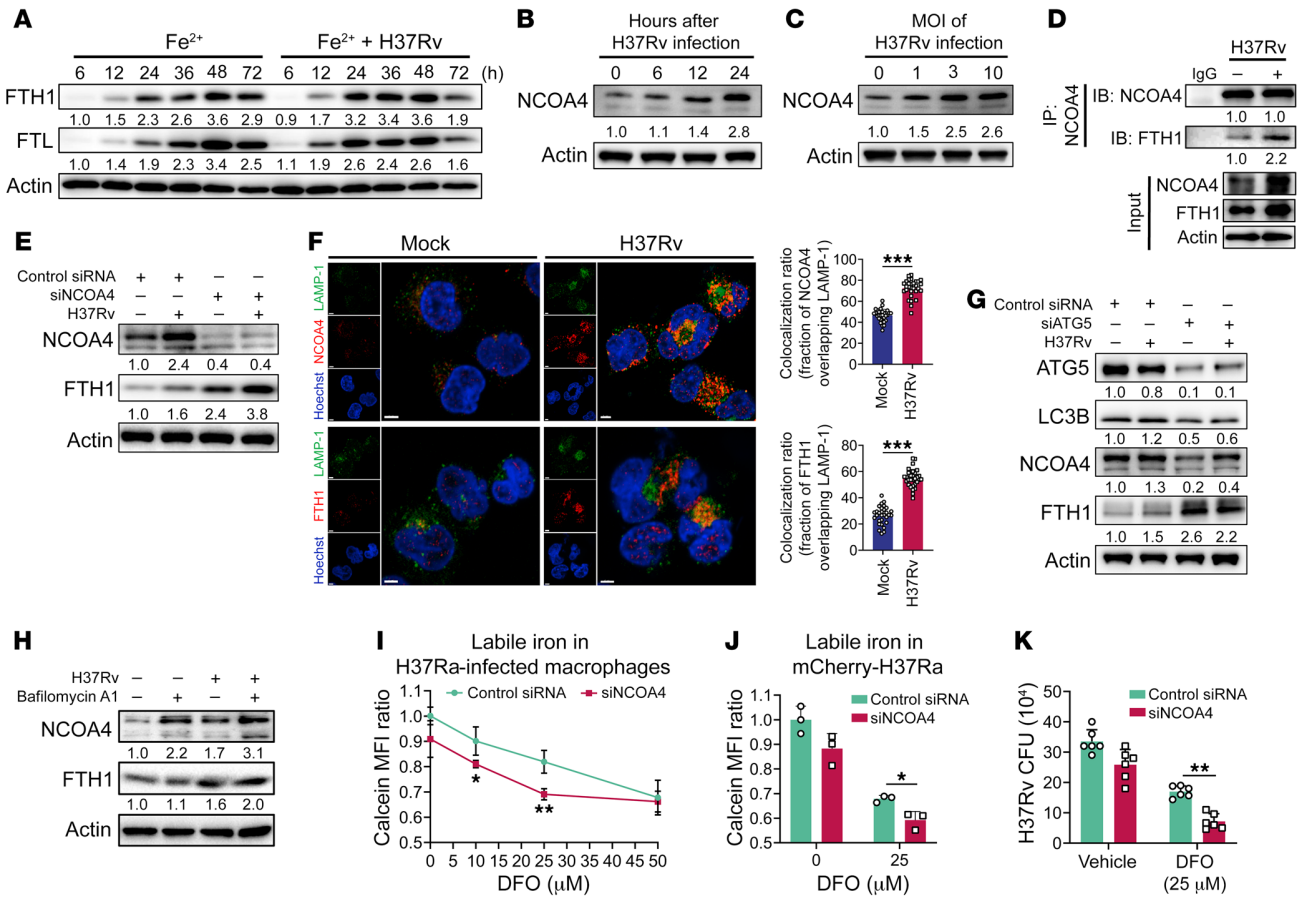
Mtb-induced ferritinophagy facilitates intracellular bacterial growth by enabling access to bioavailable iron. (**A**) Immunoblot analysis of FTH1 and FTL at different time points (6, 12, 24, 48, and 72 hours) in THP-1–derived macrophages after H37Rv infection in the presence of ferrous lactate (20 μM). (**B** and **C**) Immunoblot analysis of NCOA4 at different time points (0, 6, 12, and 24 hours; **B**) in THP-1–derived macrophages after H37Rv infection with the indicated MOI (0, 1, 3, and 10; **C**). (**D**) Immunoblot analysis and immunoprecipitation of NCOA4 and FTH1 in THP-1–derived macrophages infected with H37Rv (MOI = 3) for 24 hours. (**E**) Immunoblot analysis of NCOA4 and FTH1 in NCOA4-knockdown THP-1–derived macrophages infected with H37Rv for 24 hours. (**F**) Representative images of H37Rv-infected THP-1–derived macrophages stained with FTH1, NCOA4, and LAMP-1 (scale bars: 4 μm). The colocalization ratio was quantified from 30 macrophages (right). (**G**) Immunoblot analysis of ATG5, LC3B, NCOA4, and FTH1 in AGT5-knockdown THP-1–derived macrophages infected with H37Rv for 24 hours. (**H**) Immunoblot analysis of NCOA4 and FTH1 in THP-1–derived macrophages infected with H37Rv for 24 hours, followed by bafilomycin A1 (100 nM) treatment for a further 3 hours. (**I**–**K**) Detection of free iron in NCOA4-knockdown THP-1–derived macrophages (**I**) and in mCherry^+^ H37Ra isolated from the indicated macrophages (**J**) by Calcein-AM or H37Rv CFU (**K**) in the presence or absence of various concentrations of DFO (10, 25, and 50 μM) after infection for 72 hours. Data in **D**, **E**, and **H**–**K** are representative of 2 or 3 independent experiments. Data are presented as means ± SD; *P < 0.05, **P < 0.01, ***P < 0.001 by Student’s unpaired *t* test (**F**) or 2-tailed unpaired *t* test (**I**–**K**).

Our finding that ferritin facilitated intracellular Mtb growth in macrophages cultured in iron-depleted medium prompted us to investigate whether and how Mtb hijacks ferritin metabolism for its growth. To this end, we monitored temporal changes in FTH1 and FTL levels in macrophages following the addition of iron in the presence or absence of Mtb. While induction and degradation of FTH1 and FTL were largely driven by the presence of iron, Mtb infection accelerated ferritin turnover induced by iron (Figure 2A). Accelerated FTH1 and FTL degradation were also confirmed in ferritin-preloaded macrophages (Supplemental Figure 2A). Ferritin degradation results in iron release, a process known as ferritinophagy (15, 16), through which NCOA4 functions as a selective autophagic receptor for the uptake and trafficking of ferritin into lysosomes (15, 17). Consequently, we examined NCOA4-mediated ferritinophagy in Mtb-infected macrophages. We observed that the levels of NCOA4 were upregulated in a time- and dose-dependent manner upon Mtb infection (Figure 2, B and C). NCOA4 was found to interact directly with FTH1 in Mtb-infected macrophages (Figure 2D and Supplemental Figure 2B), while the knockdown of NCOA4 prevented ferritin degradation (Figure 2E) without affecting cell death of Mtb-infected macrophages (Supplemental Figure 2C). Immunofluorescence staining revealed that the expression of both NCOA4 and FTH1 was enhanced following Mtb infection and that NCOA4 and FTH1 colocalized within lysosomes (Figure 2F and Supplemental Figure 2, D and E). Inhibition of autophagy by silencing of ATG5 or intervening with autophagosome-lysosome fusion and acidification using chloroquine and bafilomycin A1 resulted in ferritin accumulation (Figure 2, G and H, and Supplemental Figure 2F). Together, these results indicate that Mtb infection accelerates ferritin degradation via enhancing NCOA4-mediated ferritinophagy.

We next sought to determine whether enhanced ferritinophagy facilitates intracellular Mtb growth by increasing iron availability. To this end, we used Calcein-AM, an iron-specific fluorescent dye, to measure the level of intracellular free iron, as previously described (Supplemental Figure 2G) (18–20). As expected, intracellular free iron was significantly decreased in Mtb-infected NCOA4-knockdown macrophages in the presence of 10 or 25 μM DFO (Figure 2I). We validated the reduced intracellular free iron in Mtb-infected NCOA4-knockdown macrophages using another free iron–specific fluorescent dye, FeRhoNox (Supplemental Figure 2H) (21, 22). In concordance with the decrease in intracellular free iron, Mtb isolated from NCOA4-knockdown macrophages exhibited increased calcein fluorescence, indicating decreased iron acquisition by Mtb (Figure 2J). Accordingly, intracellular Mtb growth was significantly suppressed in NCOA4-knockdown macrophages (Figure 2K and Supplemental Figure 2I), while there was no significant difference in macrophages with double NCOA4 and FTH1 knockdown (Supplemental Figure 2J). Taken together, these findings indicate that NCOA4-mediated ferritinophagy is essential for intracellular Mtb growth by increasing the iron accessible to these bacteria.

### Reduced FTH1 in macrophages is associated with the progression of human TB.

**Figure 3:**
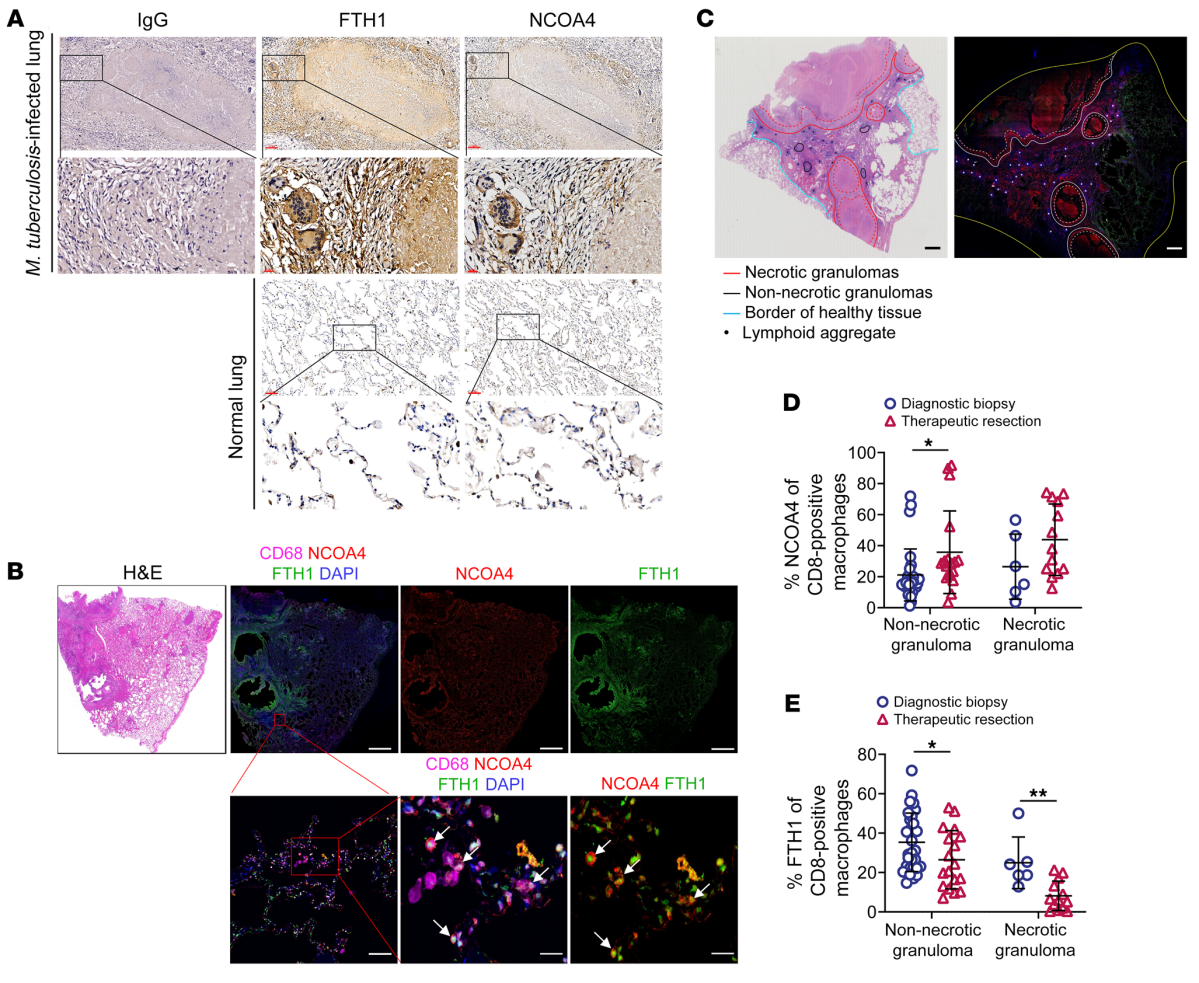
Reduced FTH1 in macrophages is associated with the progression of human TB. (**A**) Immunohistochemical staining for NCOA4 and FTH1 in lung tissue from a TB patient or a normal lung. Scale bars: 100 μm (top) and 20 μm (bottom). (**B**) Colocalization of NCOA4 and FTH1 in CD68-positive macrophages in the lung lesions of TB patients. Top: H&E staining, single-channel images, and a merged image of DAPI, CD68, NCOA4, and FTH1 (scale bars: 2 mm). Bottom: Magnifications of the outlined areas (scale bars: 100 μm). The arrows denote the colocalization of NCOA4 and FTH1 in CD68-positive macrophages. (**C**–**E**) Immunofluorescence staining of lung tissues from TB patients undergoing therapeutic resection (*n* = 9) and diagnostic biopsy specimens (*n* = 6). (**C**) Representative H&E image and immunofluorescence image of a therapeutic resection tissue section with various necrotizing and non-necrotizing granulomas and lymphoid aggregate (scale bars: 2 mm). (**D** and **E**) Quantification of the percentage of NCOA4-positive (**D**) and FTH1-positive (**E**) macrophages in well-organized necrotizing and non-necrotizing granulomas among 2 cohorts. A total of 48 non-necrotizing granulomas (18 from therapeutic resection and 30 from diagnostic biopsy specimens) and 19 necrotizing granulomas (13 from therapeutic resections and 6 from diagnostic biopsy specimens) were included in the analysis. Data are presented as means ± SD; *P < 0.05, **P < 0.01 by Student’s 2-tailed unpaired *t* test (**D** and **E**).

To investigate the clinical relevance of increased ferritinophagy in human TB, we first examined ferritinophagy in normal and tuberculous lung tissues. Both NCOA4 and FTH1 were highly expressed in cells with morphologic characteristics of macrophages surrounding tuberculous granulomas in human lungs (Figure 3A). In addition, high levels of FTH1 and NCOA4 were observed in macrophages colocalized within TB lesions (Figure 3B).

Next, we aimed to investigate the potential association between ferritinophagy and progression of human TB. For this, we obtained pulmonary tissues from patients undergoing therapeutic resection for advanced TB (*n* = 9) and diagnostic biopsy specimens for earlier-stage TB (*n* = 6) and subsequently compared the expression of NCOA4 and FTH1 using quantitative immunohistochemistry (Supplemental Table 1). Across all pulmonary tissues, we observed that the well-recognized necrotizing granulomas were frequently accompanied by non-necrotizing granulomas and lymphoid aggregates, as previously described (Figure 3C) (23). By quantifying the percentages of NCOA4-positive macrophages within non-necrotizing and necrotizing granulomas, we observed a higher percentage of NCOA4-positive macrophages in the therapeutic resection tissues than in the diagnostic biopsy specimens. However, the difference was statistically significant only in non-necrotizing granulomas (Figure 3D). In contrast, the percentages of FTH1-positive macrophages were significantly higher in the diagnostic biopsy specimens than in the therapeutic resection tissues in non-necrotizing and necrotizing granulomas (Figure 3E). Therefore, we suggest that the upregulation of FTH1 in macrophages is associated with TB disease progression in humans.

### Mtb infection increases NCOA4 expression by regulating TRIM21-mediated proteasomal degradation of HERC2.

**Figure 4:**
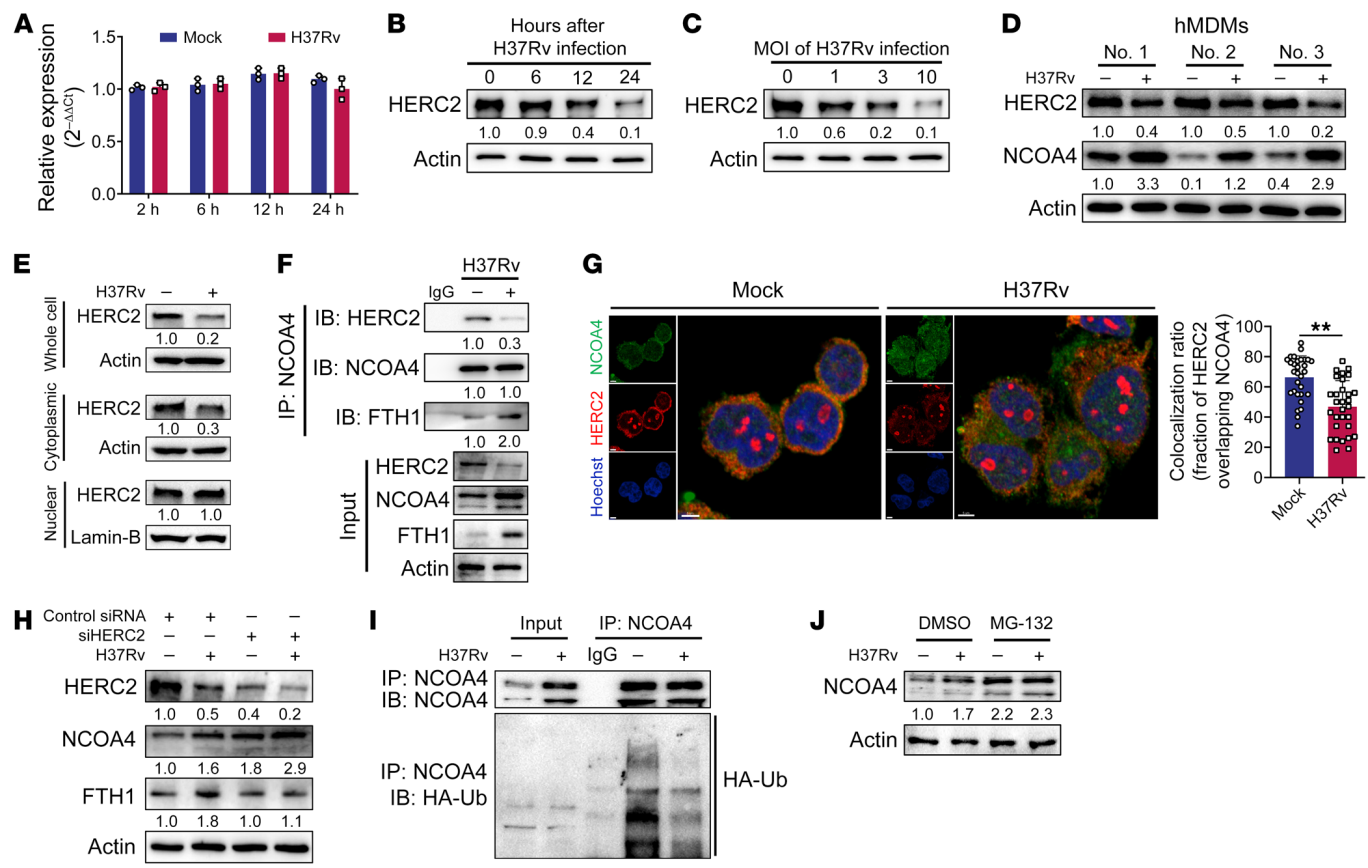
Mtb-induced NCOA4 is regulated by HERC2-mediated proteolysis. (**A**) RT-qPCR analysis of *NCOA4* in THP-1–derived macrophages infected with H37Rv. (**B** and **C**) Immunoblot analysis of NCOA4 in THP-1–derived macrophages infected with H37Rv (MOI = 1, 3, and 10; **C**) for 2, 6, 12, and 24 hours (**B**). (**D**) Immunoblot analysis of HERC2 and NCOA4 in human monocyte-derived macrophages (hMDMs; *n* = 3) infected with H37Rv for 24 hours. (**E**) Immunoblot analysis of HERC2 in the cytoplasmic and nuclear fractions of control and H37Rv-infected THP-1–derived macrophages. (**F**) Immunoblot analysis and immunoprecipitation of NCOA4, HERC2, and FTH1 in THP-1–derived macrophages infected with H37Rv for 24 hours. (**G**) Representative images of H37Rv-infected THP-1–derived macrophages stained with HERC2 and NCOA4 (scale bars: 5 μm). The colocalization ratio was quantified from 30 macrophages (right). (**H**) Immunoblot analysis of HERC2, NCOA4, and FTH1 in THP-1–derived macrophages transfected with HERC2 siRNA and then infected with H37Rv for 24 hours. (**I**) Immunoblot analysis and immunoprecipitation of NCOA4 and HA-Ub in THP-1–derived macrophages transfected with HA-Ub plasmid followed by H37Rv infection for 24 hours. (**J**) Immunoblot analysis of NCOA4 in THP-1–derived macrophages infected or not infected with H37Rv for 24 hours, followed by MG-132 (10 μM) treatment for a further 3 hours. Data in **A**, **E**, **F**, **H**, and **J** are representative of 2 or 3 independent experiments. Data are presented as means ± SD; **P < 0.01 by Student’s unpaired *t* test (**G**).

The results described above point to increased NCOA4 as a key event in the degradation of ferritin and release of iron for Mtb growth. Nevertheless, Mtb infection did not affect the mRNA level of *NCOA4* (Figure 4A). It has been recognized that induction of ferritinophagy via NCOA4 is regulated by HERC2-mediated proteolysis during erythropoiesis (15, 24). We, therefore, investigated whether HERC2 expression in macrophages was reduced during Mtb infection. As expected, the level of HERC2 in THP-1 macrophages decreased following Mtb infection in a time- and dose-dependent manner (Figure 4, B and C). Notably, the Mtb infection–induced decrease in HERC2 expression was recapitulated in primary human monocyte-derived macrophages, concordant with increased NCOA4 expression (Figure 4D).

HERC2 has been reported to reside in both the cytoplasm and nucleus (25, 26). By separating the cytoplasmic and nuclear fractions of macrophages, we found that Mtb infection reduced the level of HERC2 protein in the cytoplasm but not the nucleus (Figure 4E and Supplemental Figure 3A). Coimmunoprecipitation and confocal microscopy analysis demonstrated that Mtb infection decreased direct interaction between HERC2 and NCOA4 and simultaneously increased the amount of FTH1 protein bound to NCOA4 in macrophages (Figure 4, F and G, and Supplemental Figure 3B). We further validated the negative regulation by HERC2 of NCOA4-mediated ferritin degradation. HERC2 knockdown increased the abundance of NCOA4 in both control and Mtb-infected macrophages, resulting in accelerated degradation of ferritin (Figure 4H). In addition, Mtb infection inhibited NCOA4 ubiquitination in THP-1 macrophages transfected with plasmids encoding ubiquitin (HA-Ub) (Figure 4I and Supplemental Figure 3C). NCOA4 abundance increased following treatment with the proteasome inhibitor MG-132, suggesting a role for proteasomes in regulating NCOA4 during Mtb infection (Figure 4J and Supplemental Figure 3, D and E). Collectively, our data indicate that HERC2-mediated proteolysis inhibits the turnover of NCOA4 protein in Mtb-infected macrophages.

**Figure 5:**
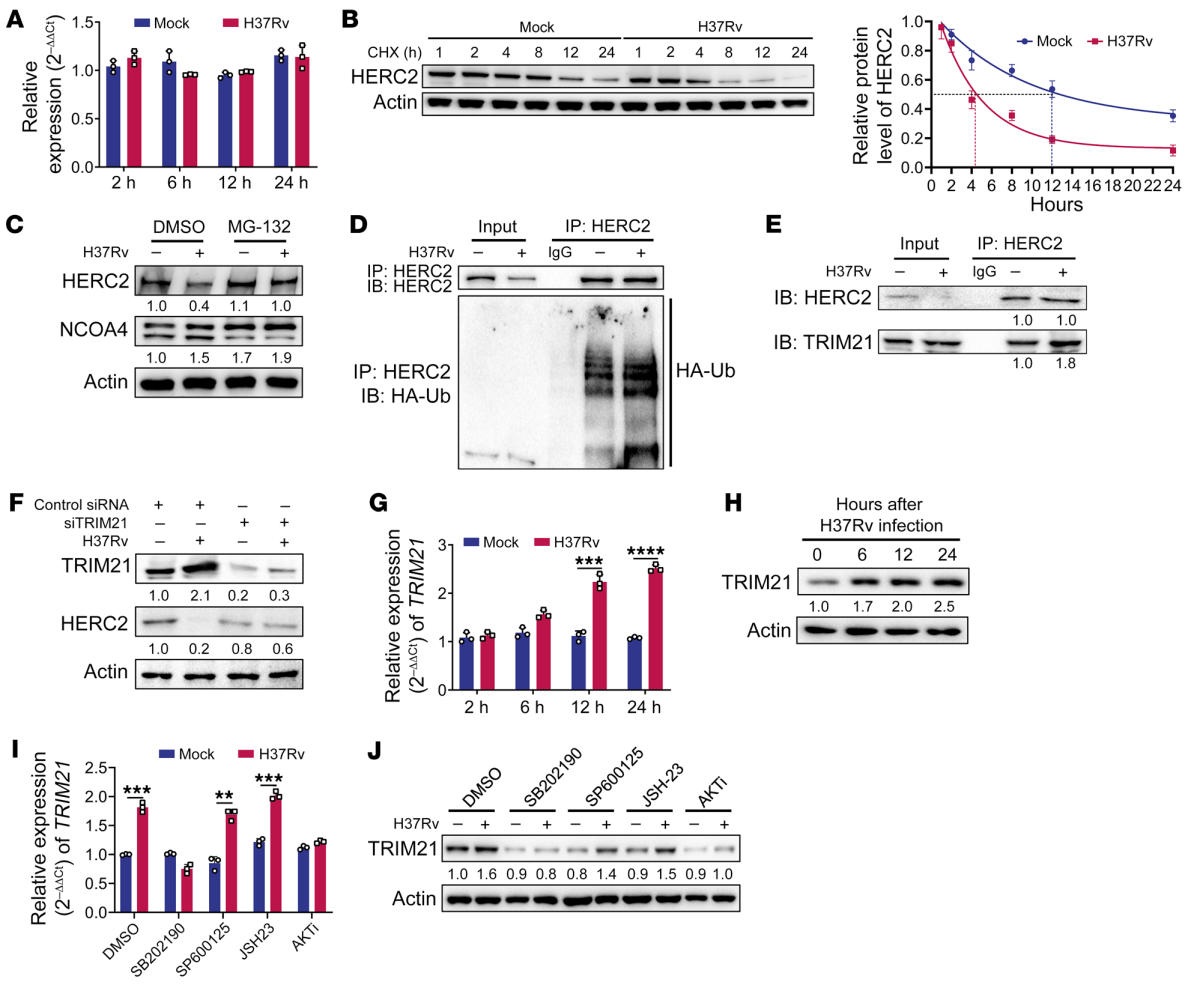
Mtb-induced proteasomal degradation of HERC2 depends on TRIM21. (**A**) RT-qPCR analysis of *HERC2* in THP-1–derived macrophages infected with H37Rv for 2, 6, 12, and 24 hours. (**B**) Immunoblot analysis and quantitative analysis of HERC2 in THP-1–derived macrophages infected with H37Rv for various times (1, 2, 4, 8, 12, and 24 hours) in the presence of cycloheximide (CHX; 10 μM). (**C**) Immunoblot analysis of HERC2 and NCOA4 in THP-1–derived macrophages infected with H37Rv for 24 hours, followed by MG-132 (10 μM) for a further 3 hours. (**D**) Immunoblot analysis and immunoprecipitation of HERC2 and HA-Ub in THP-1–derived macrophages transfected with HA-Ub plasmid, followed by H37Rv infection for 24 hours. (**E**) Immunoblot analysis and immunoprecipitation of HERC2 and TRIM21 in THP-1–derived macrophages infected with H37Rv for 24 hours. (**F**) Immunoblot analysis of HERC2 and TRIM21 in THP-1–derived macrophages transfected with TRIM21 siRNA and then infected with H37Rv for 24 hours. (**G** and **H**) RT-qPCR analysis (**G**) and immunoblot analysis (**H**) of TRIM21 in THP-1–derived macrophages infected with H37Rv for 0, 6, 12, and 24 hours. (**I** and **J**) RT-qPCR analysis (**I**) and immunoblot analysis (**J**) of TRIM21 in THP-1–derived macrophages infected with H37Rv for 24 hours in the presence of p38 inhibitor (SB202190, 10 μM), JNK inhibitor (SP600125, 20 μM), NF-κB inhibitor (JSH-23, 30 μM), or AKT1 inhibitor (AKTi, 10 μM). Data in **A**–**C**, **E**, **F**, **I**, and **J** are representative of 2 or 3 independent experiments. Data are presented as means ± SD; **P < 0.01, ***P < 0.001, ****P < 0.0001 by Student’s 2-tailed unpaired *t* test (**G** and **I**).

Although evidence has been presented that HERC2 functions as an E3 ligase (24), how HERC2 expression is regulated has not been investigated. Similarly to NCOA4, the transcript level of HERC2 was not influenced by Mtb infection (Figure 5A), suggesting that HERC2 is regulated posttranslationally. By using cycloheximide, a pan-inhibitor of protein synthesis, we found that HERC2 turnover was significantly accelerated in Mtb-infected macrophages, with the half-life of HERC2 reduced from 12 to 4 hours (Figure 5B). Additionally, inhibition of HERC2 induced by Mtb infection could be rescued by the proteasome inhibitor MG-132 (Figure 5C), but not by the lysosome maturation inhibitors chloroquine and bafilomycin A1 (Supplemental Figure 4A). We conclude that the turnover of HERC2 depends on proteasomal degradation. Finally, we observed that Mtb infection markedly induced total ubiquitination of HERC2 in macrophages (Figure 5D and Supplemental Figure 4B). Together, these data confirm that Mtb infection enhances ubiquitin-dependent proteasomal degradation of HERC2 in macrophages.

To identify the E3 ligase responsible for HERC2 degradation, we performed a mass spectrometric analysis of the proteins coimmunoprecipitated by anti-HERC2 antibody. Four E3 ligase candidates were identified. Among them, TRIM21 had the highest score in both independent experiments (Supplemental Table 2). We next tested whether TRIM21 is the E3 ligase for HERC2. The physical interaction between endogenous TRIM21 and HERC2 in macrophages during Mtb infection was confirmed by coimmunoprecipitation and confocal microscopy analysis (Figure 5E and Supplemental Figure 4, C and D). Moreover, degradation of HERC2 induced by Mtb infection was effectively prevented by TRIM21 knockdown in macrophages (Figure 5F and Supplemental Figure 4E). These findings identify the E3 ligase TRIM21 as responsible for the proteasomal degradation of HERC2 in macrophages during Mtb infection.

We next assessed the impact of Mtb infection on the E3 ligase TRIM21. Both the mRNA and protein levels of TRIM21 were upregulated in a time-dependent manner during Mtb infection (Figure 5, G and H). To further elucidate the signaling pathways underlying modulation of TRIM21 by Mtb in macrophages, we infected THP-1 macrophages with Mtb in the absence or presence of the p38 inhibitor SB202190, the JNK inhibitor SP600125, the NF-κB inhibitor JSH-23, or AKT1 inhibitor (AKTi), and then determined the mRNA and protein levels of TRIM21. Intriguingly, the induction of TRIM21 was markedly inhibited by SB202190 and AKTi (Figure 5, I and J). Thus, we propose that Mtb infection induces TRIM21 expression in macrophages mainly through p38 and AKT1 signaling pathways.

### Ncoa4 deficiency in myeloid cells increases host resistance to Mtb infection.

**Figure 6:**
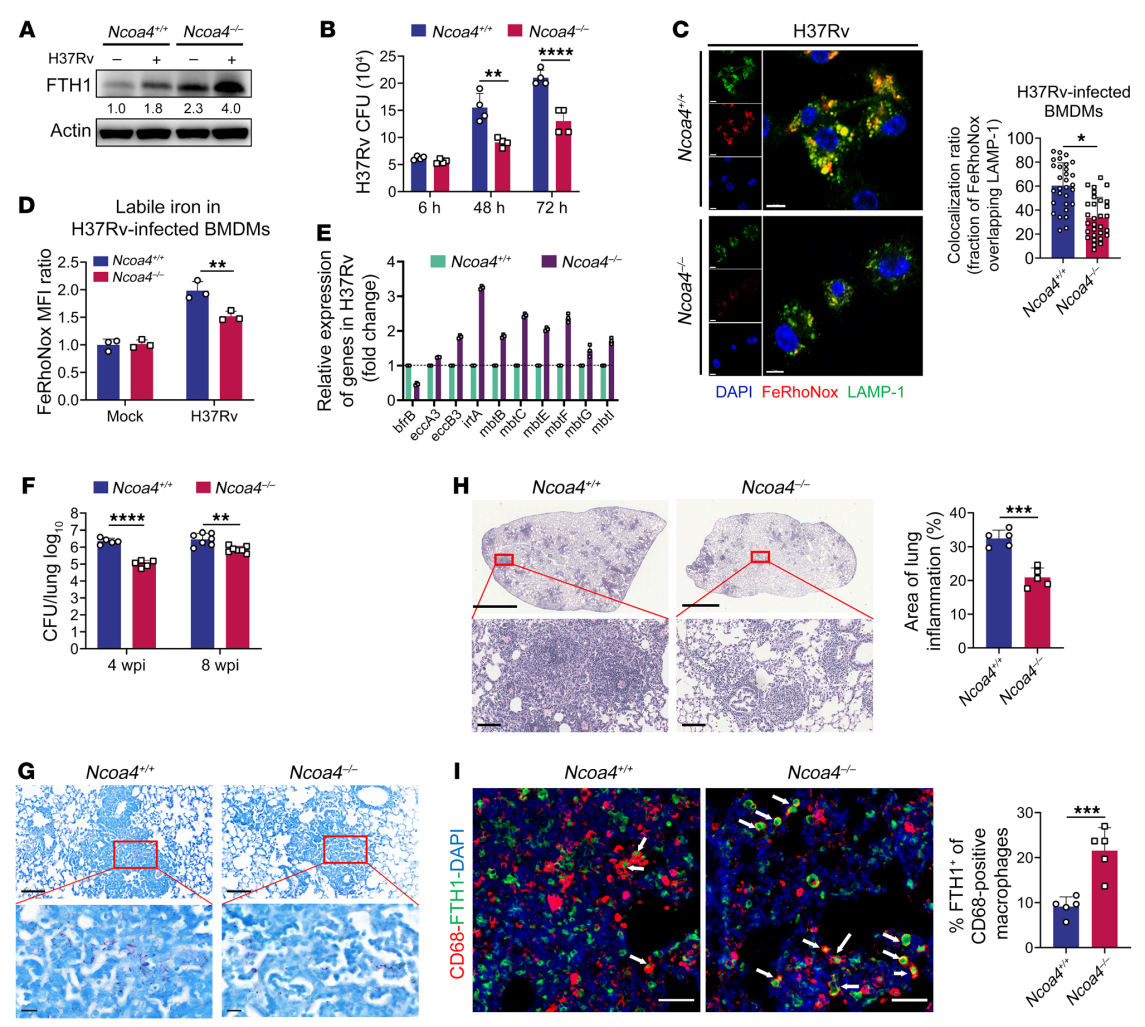
*Ncoa4* deficiency in myeloid cells increases host resistance to Mtb. (**A**) Immunoblot analysis of FTH1 in BMDMs from *Ncoa4^+/+^* and *Ncoa4^–/–^* mice infected with H37Rv for 24 hours. (**B**) In vitro CFUs in BMDMs from *Ncoa4^+/+^* and N*coa4^–/–^* mice. (**C**) Representative images of H37Rv-infected BMDMs stained with FeRhoNox and LAMP-1 (scale bars: 7 μm). The colocalization ratio was quantified from 30 macrophages (right). (**D**) Detection of free iron in BMDMs by FeRhoNox after H37Rv infection for 72 hours. (**E**) The fold changes of iron-response genes in H37Rv isolated from the indicated BMDMs. (**F**–**I**) *Ncoa4^+/+^* and *Ncoa4^–/–^* mice were aerosol-infected with about 200 CFU per mouse of H37Rv, and lungs were removed at 4 and 8 weeks postinfection (wpi). The burden of H37Rv in the lungs of *Ncoa4^+/+^* and *Ncoa4^–/–^* mice (4 wpi, *n* = 5 per group; 8 wpi, *n* = 7 per group) was assessed by counting of CFUs (**F**) and acid-fast staining (**G**, 4 wpi; scale bars: 100 μm [top] and 20 μm [bottom]. Histopathology was assessed in lung sections stained with H&E and quantified by immune cell infiltration ratio (**H**, 4 wpi; scale bars: 2 mm [top] and 100 μm [bottom]). FTH1 (green) in macrophages (red, CD68-positive) was immunofluorescently stained (scale bars: 20 μm) and quantified by percentage (**I**; *n* = 100). Data in **A**–**D** and **F** are representative of 2 or 3 independent experiments. Data are presented as means ± SD; *P < 0.05, **P < 0.01, ***P < 0.001, ****P < 0.0001 by Student’s unpaired *t* test (**C**, **H**, and **I**) or Student’s 2-tailed unpaired *t* test (**B**, **D**, and **F**).

To investigate whether NCOA4-mediated ferritinophagy contributes to host resistance against Mtb infection in vivo, we generated conditional-*Ncoa4*-knockout mice with *Ncoa4* deficiency in myeloid cells, including monocytes/macrophages (Lyz2^cre^ *Ncoa4^fl/fl^*, hereafter referred to as *Ncoa4^–/–^*). We used PCR to confirm *Ncoa4* alleles in control littermate mice (*Ncoa4^fl/fl^*, hereafter referred to as *Ncoa4^+/+^*) and *Ncoa4^–/–^* mice (Supplemental Figure 5A). In agreement with the results obtained with the NCOA4-knockdown THP-1 macrophages, ferritinophagy was impaired in bone marrow–derived macrophages (BMDMs) from *Ncoa4^–/–^* mice, as determined by the accumulation of FTH1 (Figure 6A). Moreover, the growth of intracellular Mtb was significantly reduced in *Ncoa4^–/–^* BMDMs (Figure 6B). As expected, *Ncoa4^–/–^* BMDMs showed significantly decreased levels of free iron in lysosomes (Figure 6C) and host macrophages (Figure 6D) upon Mtb infection, which was confirmed by the changes of iron-response genes in intracellular Mtb, as previously reported (Figure 6E) (27, 28).

Following aerosol infection with Mtb (H37Rv), the bacterial loads in the lungs of *Ncoa4^–/–^* mice were significantly reduced compared with those in *Ncoa4^+/+^* mice, 4 and 8 weeks after infection (Figure 6, F and G). Consistently, tissue consolidation in the lungs of *Ncoa4^–/–^* mice was impaired (Figure 6H), indicating that NCOA4 affects host resistance to Mtb infection in vivo. Although lymphocyte, monocyte/macrophage, and neutrophil infiltration into the lungs did not differ significantly between *Ncoa4^+/+^* and *Ncoa4^–/–^* mice (Supplemental Figure 5B), the accumulation of FTH1^+^CD68^+^ macrophages in the lung tissues of infected *Ncoa4^–/–^* mice increased (Figure 6I). In addition, we found that most of the cytokines we detected, such as INF-γ, IL-1β, and TNF-α, were present at significantly higher levels in lung homogenates of *Ncoa4^–/–^* mice compared with those of *Ncoa4^+/+^* mice at 4 weeks after infection, suggesting that NCOA4 suppresses pulmonary inflammation and impairs bacterial control. Interestingly, there were no significant differences in cytokine levels between the 2 groups at 8 weeks after infection (Supplemental Figure 5C). We speculate that the NCOA4-dependent mechanism plays a more prominent role in the early stages of Mtb infection than in the later phases of the infection. In agreement with our in vitro findings, our in vivo results confirm that Mtb infection enhances NCOA4-mediated ferritinophagy, facilitating the intracellular persistence of the pathogen.

## Discussion

The immune system has evolved to protect the host against various microorganisms (29). However, several pathogens have developed survival strategies that allow them to evade ongoing immune responses (1, 2). Iron is essential for both host and pathogen, and the sequestration and release of iron by ferritin regulate iron availability, thereby preventing toxicity associated with iron overload and hindering pathogens’ access to this essential nutrient that is stored in phagolysosomes. Here, we showed that ferritin can be either detrimental or beneficial for intracellular Mtb, depending on the availability of free iron. This difference provides an alternative explanation for the discrepant results reported for the role played by FTH1 deficiency in the macrophage-mediated killing of Mtb (3, 4). More importantly, we demonstrated that Mtb exploits the autophagy-dependent ferritin degradation mechanisms of the host to promote its intracellular persistence via the increased availability of iron in lysosomes. This previously unappreciated evasion mechanism is initiated by p38 and AKT1 signaling and mediated by a TRIM21-dedendent proteasomal degradation cascade.

NCOA4 is a key regulator of ferritinophagy, a biological process that degrades ferritin and thereby releases bound iron (16, 30, 31). We demonstrated that NCOA4-mediated ferritin degradation significantly increases the availability of free iron during Mtb infection and facilitates Mtb growth. In the absence of NCOA4, silencing of FTH1 failed to enhance intracellular Mtb growth. Intriguingly, our data revealed that NCOA4 deficiency in myeloid cells significantly increased resistance to Mtb infection in mice, and NCOA4-mediated ferritin degradation correlated with disease status in patients with TB. Together, these findings demonstrate an essential role for NCOA4 in regulating the effects of ferritin on Mtb infection. In contrast to recent studies reporting that iron overload in urothelial cells induced NCOA4-dependent ferritinophagy leading to hyper-replication of *E*. *coli* and host cell death (7), and that a secreted virulence factor of *Ehrlichia chaffeensis*, Etf-3, directly bound the ferritin light chain and induced ferritinophagy to enhance intracellular growth (8), we demonstrated that Mtb infection exclusively upregulated the level of NCOA4 and accelerated ferritin degradation to release free iron for intracellular Mtb growth. Collectively, the results of these studies suggest that the exploitation of host ferritinophagy to access iron represents a fundamental evasion strategy adopted by a variety of pathogens.

Intriguingly, we identified TRIM21 as a critical link between innate signaling and iron metabolism in macrophages during Mtb infection. It is known that HERC2-mediated proteolysis of NCOA4 is required for the induction of ferritinophagy in erythropoiesis (15, 24, 32); however, how this interaction is regulated in other cell types, such as macrophages, notably in response to a microbial challenge, remained unknown. We demonstrated that Mtb infection decreased protein levels but not transcript levels of HERC2, suggesting post-transcriptional regulation of HERC2. By employing mass spectrometry, we revealed, for the first time to our knowledge, that TRIM21, an E3 ligase, is responsible for the proteolytic degradation of HERC2. TRIM21, also known as RO52, is a common target of circulating autoantibodies in autoimmune diseases (33, 34). The specific virulence factors of Mtb that manipulate the expression of TRIM21 in macrophages remain unknown. It is clear from our studies, however, that the increased TRIM21 in macrophages upon Mtb infection depends on the p38/AKT1 signaling pathway. Future investigations are needed to identify the virulence factors of Mtb responsible and to elucidate the mechanisms that initiate ferritinophagy.

Ferritinophagy has been demonstrated to accumulate free iron and promote ferroptosis, an iron- and reactive oxygen species–dependent form of regulated cell death (17, 35–37). Recently, ferroptosis has been described as a major mechanism of necrosis in Mtb-infected macrophages, which may facilitate mycobacterial spread (18). However, we did not observe any effects of NCOA4 knockdown on cell death in Mtb-infected THP-1 macrophages. Furthermore, cellular immune responses in Mtb-infected *Ncoa4^+/+^* and *Ncoa4^–/–^* mice did not differ. Further investigations are warranted to determine whether free iron from ferritin degradation serves as a nutrient or interrupts ferroptosis to benefit intracellular Mtb growth. However, our findings indicate that the ferritinophagy-mediated susceptibility to Mtb infection is independent of cell death.

While there is an association between iron dysregulation and related anemia, there is currently no effective host-directed therapeutic (HDT) strategy for TB available that targets iron metabolism (38, 39). Clinical trials have shown that iron overload is a risk factor for patients with TB and is associated with poor outcomes (40, 41), which may be the result of cytotoxicity and/or enhanced Mtb growth (42). Similarly, an association between iron deficiency and increased risk of recurrence of TB and death has been observed (43, 44). TB-associated anemia is predominantly caused by absorbed dietary iron being retained in ferritin within macrophages while circulating iron rapidly decreases following inflammation (45). However, in clinical trials and animal studies, neither iron supplementation nor iron depletion has been found to improve TB, probably because of the dual role of ferritin in Mtb infection (38, 40, 46, 47). By establishing the critical role played by NCOA4-mediated ferritinophagy in regulating the effects of ferritin on intracellular Mtb growth, we have revealed a new pathomechanism in TB, one that represents a potential HDT target for intervening with iron metabolism during TB pathogenesis.

## Methods

### Human TB granuloma cohort.

Archival formalin-fixed, paraffin-embedded (FFPE) specimens from patients undergoing therapeutic resection for advanced TB (*n* = 9) and diagnostic biopsy specimens for earlier stage TB (*n* = 6) were procured from Shenzhen Third Hospital and Shenzhen People’s Hospital. Tissues were screened to include only those that comprised epithelioid cells, multiple Langhans giant cells, and caseous necrosis or were positive for acid-fast bacilli staining, nucleic acid amplification test, and Mtb culture (48). To screen for the presence of granuloma, each specimen was stained with H&E and inspected by 2 anatomic pathology experts.

### Mice.

Lyz2^cre^ *Ncoa4^fl/fl^* (referred to as *Ncoa4^–/–^*) mice were generated on the C57BL/6 background via transgenic animal services provided by Cyagen (Jiangsu, China). Briefly, the guide RNA (gRNA) to the mouse *Ncoa4* gene, the donor vector containing *loxP* sites flanking exons 2–6, and *Cas9* mRNA were coinjected into fertilized mouse eggs to generate offspring with targeted conditional knockout. The mouse mating was performed by crossing of mice carrying the floxed *Ncoa4* allele (F_0_, *Ncoa4^fl/+^*) with mice expressing Cre recombinase (Lyz2^cre^) to generate F_1_ mice (Lyz2^cre^ *Ncoa4^fl/+^*) that were heterozygous for a targeted allele and hemizygous/heterozygous for the Cre transgene. Heterozygous, Cre^+^ mice were bred with homozygous mice. *Ncoa4^fl/fl^* mice were used as control littermate mice (referred to as *Ncoa4^+/+^*). Successful deletions were confirmed by PCR and DNA sequencing. The sequences of the gRNAs were as follows: gRNA1 (matching the forward strand of the gene), CCACTTCCAGCACTACGTAGGGG; gRNA2 (matching the reverse strand of the gene), TGCCTACAACCTAGCGCTCTGGG; gRNA3 (matching the forward strand of the gene), CTTCCAGCACTACGTAGGGGAGG; and gRNA4 (matching the reverse strand of the gene), CTACAACCTAGCGCTCTGGGAGG.

### Bacterial strains and culture.

The mycobacterial strains, including the virulent and attenuated *M*. *tuberculosis* (Mtb) strains H37Rv and H37Ra, respectively, were cultured either in Middlebrook 7H9 (BD Difco) broth supplemented with 0.2% (vol/vol) glycerol, 0.25% (vol/vol) Tween-80 (MilliporeSigma), and 10% OADC (BD BBL) or on Middlebrook 7H10 (BD Difco) plates supplemented with 0.5% glycerol and 10% OADC.

### Cell isolation and culture.

PBMC isolation and human monocyte-derived macrophage (hMDM) and THP-1 macrophage differentiation were performed as previously described (49). Briefly, PBMCs were obtained from whole blood by density gradient centrifugation (400*g*, 30 minutes) and then incubated overnight to enrich for monocytes by adherence to plastic culture plates. The adherent monocytes were differentiated into hMDMs by treatment with 50 ng/mL of human macrophage colony-stimulating factor (PeproTech) for 5 days. Human monocytic THP-1 (TIB-202, ATCC) cells were plated at 4 × 10^5^ cells/mL in 6- or 12-well plates (Costar) and treated with 40 ng/mL PMA (Sigma-Aldrich) for 48 hours to differentiate into macrophages. BMDMs were isolated from *Ncoa4^+/+^* or *Ncoa4^–/–^* mice as previously described (50). Bone marrow cells were cultured in DMEM supplemented with 20% conditioned medium from L929 cells, 1 mM sodium pyruvate, 2 mM l-glutamine, and 10% FBS for 5–7 days. PMA-differentiated THP-1 macrophages, hMDMs, and BMDMs were maintained in fresh, prewarmed media until further use.

### Macrophage infection model.

The PMA-differentiated human THP-1 macrophages, hMDMs, or BMDMs were infected with Mtb H37Rv (multiplicity of infection [MOI] = 3) or H37Ra (MOI = 10) for 6 hours and then washed 3 times. The cells were treated with DFO in the range of 10–50 μM for a further 72 hours. Subsequently, the cells were lysed with 0.1% SDS, and serial dilutions were plated on 7H10 plates. The numbers of colony-forming units (CFU) were counted after incubation at 37°C for 2–4 weeks, as previously described (49). For flow cytometry sorting, the PMA-differentiated THP-1 macrophages were infected with the Mtb strain H37Ra carrying a dual-color reporter that comprised a constitutively expressed (Emerald, green) and a tetracycline-inducible (TagRFP, red) fluorescent protein. After infection for 3 days, 500 ng/mL tetracycline (MedChemExpress) was added to the culture medium for a further 24 hours, and then macrophages were harvested for sorting on a BD FACSAria II (BD Biosciences), as previously described (13).

### Intracellular and bacterial iron quantification.

The intracellular free iron assay was performed as previously described (18, 20, 22). A total of 4 × 10^5^ PMA-differentiated THP-1 macrophages were cultured in 12-well plates (Costar) and infected or not infected with Mtb H37Rv (MOI = 3) and H37Ra (MOI = 10) for 6 hours. The macrophages were washed 3 times with PBS to remove any noninternalized bacteria and incubated in fresh complete medium with or without DFO in the range of 10–50 μM for a further 72 hours. The macrophages were washed twice with PBS and stained with 500 μL 0.5 μM Calcein-AM (BioLegend) or 5 μM FeRhoNox (Goryo Chemical) in PBS for 30 minutes at 37°C. After rinsing twice, the median fluorescence intensity of calcein or FeRhoNox in the macrophages was immediately detected using a BD FACSAria II flow cytometer (BD Biosciences) and analyzed using FlowJo software version 10 (BD Biosciences). A calcein-based bacterial free iron assay was performed using a slightly modified protocol that was previously described (20). A total of 1 × 10^6^ PMA-differentiated THP-1 macrophages were cultivated in 6-well plates (Costar) and infected or not infected with mCherry-H37Ra (MOI = 10) for 6 hours. The macrophages were washed 3 times with PBS to remove any noninternalized bacteria and incubated in fresh complete medium with or without 25 μM DFO for a further 72 hours. At the end of the infection period, the macrophages were washed 3 times with PBS to remove any extracellular bacteria, and the cells were lysed with 1 mL 0.005% SDS in PBS on ice for 15 minutes. Lysates were harvested and pelleted by centrifugation at 21,000*g* at 4°C for 20 minutes. The supernatants were carefully and completely removed, and the bacterial pellet was stained in 20 μL of 1 mM Calcein-AM in PBS in a thermomixer for 2.5 hours at 36°C and 1,500*g*. The bacterial pellet was washed twice with PBS, counted using a BD FACSAria II flow cytometer (BD Biosciences), and analyzed using FlowJo software version 10 (BD Biosciences). Bacteria could be identified by the expression of the mCherry marker.

### Plasmids, siRNAs, and transfection.

Before plasmid transfection, PMA-differentiated THP-1 macrophages were treated for 30 minutes with NATE (InvivoGen; 100×), a nucleic acid transfection enhancer, to boost transfection efficiency in macrophages (51). Then the macrophages were transfected with plasmids encoding wild-type ubiquitin (HA-Ub, a gift from Xingzhi Xu, 34142045). At 48 hours after transfection, the macrophages were infected with H37Ra (MOI = 10) for 24 hours. The supernatants of the cell lysates were subjected to immunoprecipitation and then analyzed by immunoblotting. For siRNA transfection, *FTH1* (RiboBio; stB0006367B-1-5), *FTL* (RiboBio; stB0006368B-1-5), *NCOA4* (RiboBio; stB0003663B-1-5), *ATG5* (RiboBio; siB12531154855-1-5), *HERC2* (5′-GAGCUGAUUUCUUGAGUAA-3′), and *TRIM21* (5′-GTGAAGCAGCCTCCTTATA-3′) siRNAs were transfected using Lipofectamine RNAiMAX (Invitrogen), according to the manufacturer’s protocol and as previously described (49). Scrambled siRNA was used as a negative control. At 36–48 hours after transfection, the efficiency of knockdown was determined by Western blotting.

### RNA extraction and quantitative PCR.

Total RNA was extracted from PBMCs or macrophages using an RNeasy kit (Omega), and quantitative PCR was performed using a 7500 Fast Real-Time PCR System (Thermo Fisher Scientific) with SYBR Green PCR Master Mix (TaKaRa), according to the manufacturer’s instructions and as previously described (13, 52). The primers obtained from the PrimerBank were as follows: *FTH1* (forward: 5′-AGAACTACCACCAGGACTCA-3′; reverse: 5′-TCATCGCGGTCAAAGTAGTAAG-3′), *FTL* (forward: 5′-CAGCCTGGTCAATTTGTACCT-3′; reverse: 5′-GCCAATTCGCGGAAGAAGTG-3′), *NCOA4* (forward: 5′-GAGGTGTAGTGATGCACGGAG-3′; reverse: 5′-GACGGCTTATGCAACTGTGAA-3′), *HERC2* (forward: 5′-TCGCCTCGACTCCAAATGG-3′; reverse: 5′-TCTTTGTTCCACTTGGTTCGAC-3′), and *TRIM21* (forward: 5′-TCAGCAGCACGCTTGACAAT-3′; reverse: 5′-GGCCACACTCGATGCTCAC -3′).

### Immunoprecipitation, immunoblotting, and mass spectrometry.

Cell lysates were harvested after treatment in NETN buffer containing 150 mM NaCl, 20 mM Tris-HCl (pH 7.5), 1 mM EDTA, 0.5% Nonidet P-40, and a protease inhibitor cocktail (Roche), as previously described (13). Antibodies and control IgG (1 μg) were used for immunoprecipitation from 200 μg of total lysates and incubated at 4°C overnight. Precipitates were washed 3 times with NETN buffer and quantified on a Qubit 4.0 Fluorometer using a Qubit protein assay kit (Thermo Fisher Scientific). The samples were separated by SDS-PAGE and transferred onto PVDF membranes (Merck Millipore). After blocking with 5% skim milk in PBST (PBS with 0.05% Tween-20) for 2 hours at room temperature, the membranes were incubated with primary antibodies against target proteins overnight at 4°C. After washing with PBST, the membranes were incubated with peroxidase-conjugated secondary antibodies for 1 hour at room temperature and visualized using ECL detection solution (Thermo Fisher Scientific), as previously described (49). For the detection of ubiquitinated proteins, immunoprecipitation was carried out under denaturing conditions. For mass spectrometry, the precipitates immunoprecipitated with anti-HERC2 antibody or control IgG were eluted using an elution buffer (0.5 mol/L NH_4_OH, 0.5 mmol/L EDTA), according to the manufacturer’s instructions. Samples were concentrated using an evaporator and subjected to liquid chromatography–mass spectrometry analyses. The following antibodies (clone number, source) were used in this study: FTH1 (polyclonal, Abcam; B-12, Santa Cruz Biotechnology), FTL (polyclonal, Proteintech), actin (EPR16769, Abcam), NCOA4 (polyclonal, Abcam; 439CT10.4.4, Invitrogen), LAMP-1 (H3A3, Abcam), ATG5 (D5G3, Cell Signaling Technology), LC3B (polyclonal, Cell Signaling Technology), CD68 (C68/684, Abcam), HERC2 (polyclonal, Abcam; polyclonal, Bethyl Laboratories Inc.; 17/HERC2, BD Transduction Laboratories), TRIM21 (polyclonal, Abcam), HA-Tag (6E2, Cell Signaling Technology), anti-rabbit Alexa Fluor 488 or 555 (polyclonal, Thermo Fisher Scientific), anti-mouse Alexa Fluor 488 or 555 (polyclonal, Thermo Fisher Scientific), and HRP-linked anti-rabbit and anti-mouse IgG (polyclonal, Abcam).

### Mouse infection model.

All work with mice was carried out in conformity with the guidelines of the Institutional Animal Committee of Shenzhen University School of Medicine. Six- to eight-week-old *Ncoa4^–/–^* and *Ncoa4^+/+^* mice were randomly divided into cages and infected with approximately 100–200 CFU of H37Rv strain using a Glas-Col inhalation exposure system, as previously described (13). Mice were euthanized at 0, 28, or 56 days after infection. Lungs were aseptically excised, homogenized, and subsequently plated on 7H11-OADC agar using a 10-fold serial dilution. CFUs were counted after 2–3 weeks of incubation at 37°C in 5% CO_2_.

### Histological analysis and acid-fast staining.

Segments of lung tissues were fixed in 10% paraformaldehyde in PBS and embedded in paraffin. Tissues were dissected into 4-mm-thick sections and stained with H&E or Ziehl-Neelsen stain (for acid-fast bacilli), according to standard protocols. Images of the whole microscopy slides were captured using a NanoZoomer digital pathology system (Hamamatsu Photonics), as previously described (13).

### Immunohistochemistry, immunofluorescence staining, and quantitative analysis.

Serial 4-mm sections of FFPE lung tissues from mice as well as human patients with TB who had undergone surgery were used for immunohistochemistry (IHC) staining using the EXPOSE rabbit-specific HRP/DAB detection IHC kit (Abcam), according to the manufacturer’s protocol. Images were captured using a NanoZoomer digital pathology system (Hamamatsu Photonics). For immunofluorescence staining, sections were processed by rounds of sequential antibody staining, detection, and stripping using the TSA-RM 4-color fIHC kit (Panovue), according to the manual provided. Briefly, sections were incubated with primary antibodies against target proteins for 1 hour at room temperature and then washed 3 times with TBST (TBS with 0.05% Tween-20). Antibody detection was performed using HRP-conjugated secondary antibody systems coupled with tyramide signal amplification (TSA) reactions (Panovue). Antibody stripping was performed by microwave treatment in pH 6 citrate buffer for 15 minutes at 95°C, followed by another round of staining. Whole slides were visualized, and images were captured using a Keyence BZ-810 fluorescence microscope with a ×20 objective. Immunofluorescence staining was correlated with H&E staining of the serial tissue sections for granuloma classification (23). As previously described (53), the number and percentage of positive pixels of the proteins of interest were counted in both well-organized necrotizing and non-necrotizing granulomas among our cohorts.

### Statistics.

Statistical analyses were performed using GraphPad Prism version 8 (GraphPad Software Inc.). Statistical significance of differences between groups was determined using a Student’s unpaired *t* test, Student’s 2-tailed unpaired *t* test, 1-way ANOVA with Tukey’s post hoc test, or 2-way ANOVA with Bonferroni’s post hoc test. Differences were considered to be significant at *P* less than 0.05. Statistical details of the experiments can be found in the figure legends.

### Study approval.

The present study was approved by the Ethics Committees of Shenzhen University, Shenzhen Third Hospital, and Shenzhen People’s Hospital (Shenzhen, China). Written informed consent was provided by all participants. The animal experimental protocols were approved by the Animal Research Ethics Committee of Beijing Chest Hospital (Beijing, China).

## Author contributions

YD, CZ, WX, KH, XW, HZ, BP, XL, and DL performed experiments and analyzed data. CS and YG analyzed quantitative immunohistochemistry data. YD, XC, YC, CHL, and BG designed experiments and discussed strategies. YD, XC, CGF, and SHEK interpreted the data and wrote the manuscript. YC and XC conceived the project. All authors read and approved the final manuscript.

## Supplementary Material

Supplemental data

## Version 1 04/17/2023

Electronic publication

## References

[1] Schaible UE, Kaufmann SHE (2004). Iron and microbial infection. Nat Rev Microbiol.

[2] Chao A (2019). Iron acquisition in *Mycobacterium tuberculosis*. Chem Rev.

[3] Reddy VP (2018). Ferritin H deficiency in myeloid compartments dysregulates host energy metabolism and increases susceptibility to *Mycobacterium tuberculosis* infection. Front Immunol.

[4] Khan N (2020). *M*. *tuberculosis* reprograms hematopoietic stem cells to limit myelopoiesis and impair trained immunity. Cell.

[5] Haschka D (2021). Ferritin H deficiency deteriorates cellular iron handling and worsens *Salmonella typhimurium* infection by triggering hyperinflammation. JCI Insight.

[6] Haschka D (2021). Iron in immune cell function and host defense. Semin Cell Dev Biol.

[7] Bauckman KA, Mysorekar IU (2016). Ferritinophagy drives uropathogenic *Escherichia coli* persistence in bladder epithelial cells. Autophagy.

[8] Yan Q (2021). Iron robbery by intracellular pathogen via bacterial effector-induced ferritinophagy. Proc Natl Acad Sci U S A.

[9] Kurthkoti K (2017). The capacity of *Mycobacterium tuberculosis* to survive iron starvation might enable it to persist in iron-deprived microenvironments of human granulomas. mBio.

[10] Pisu D (2020). Dual RNA-Seq of Mtb-infected macrophages in vivo reveals ontologically distinct host-pathogen interactions. Cell Rep.

[11] Dai Y (2019). Biomarkers of iron metabolism facilitate clinical diagnosis in *Mycobacterium tuberculosis* infection. Thorax.

[12] Visser A, van de Vyver A (2011). Severe hyperferritinemia in Mycobacteria tuberculosis infection. Clin Infect Dis.

[13] Yang Q (2019). CD157 confers host resistance to *Mycobacterium tuberculosis* via TLR2-CD157-PKCzeta-induced reactive oxygen species production. mBio.

[14] Silva-Gomes S (2013). Iron in intracellular infection: to provide or to deprive?. Front Cell Infect Microbiol.

[15] Mancias JD (2014). Quantitative proteomics identifies NCOA4 as the cargo receptor mediating ferritinophagy. Nature.

[16] Dowdle WE (2014). Selective VPS34 inhibitor blocks autophagy and uncovers a role for NCOA4 in ferritin degradation and iron homeostasis in vivo. Nat Cell Biol.

[17] Hou W (2016). Autophagy promotes ferroptosis by degradation of ferritin. Autophagy.

[18] Amaral EP (2019). A major role for ferroptosis in *Mycobacterium tuberculosis*-induced cell death and tissue necrosis. J Exp Med.

[19] Ma Y (2015). Iron-sensitive fluorescent probes: monitoring intracellular iron pools. Metallomics.

[20] Riedelberger M (2020). Type I interferon response dysregulates host iron homeostasis and enhances candida glabrata infection. Cell Host Microbe.

[21] Jin X (2021). Hinokitiol chelates intracellular iron to retard fungal growth by disturbing mitochondrial respiration. J Adv Res.

[22] Warpsinski G (2020). Nrf2-regulated redox signaling in brain endothelial cells adapted to physiological oxygen levels: consequences for sulforaphane mediated protection against hypoxia-reoxygenation. Redox Biol.

[24] Mancias JD (2015). Ferritinophagy via NCOA4 is required for erythropoiesis and is regulated by iron dependent HERC2-mediated proteolysis. Elife.

[25] Wu W (2010). HERC2 is an E3 ligase that targets BRCA1 for degradation. Cancer Res.

[26] Kang TH (2010). Circadian control of XPA and excision repair of cisplatin-DNA damage by cryptochrome and HERC2 ubiquitin ligase. Proc Natl Acad Sci U S A.

[27] Timm J (2003). Differential expression of iron-, carbon-, and oxygen-responsive mycobacterial genes in the lungs of chronically infected mice and tuberculosis patients. Proc Natl Acad Sci U S A.

[28] Pisu D (2020). Dual RNA-Seq of Mtb-infected macrophages in vivo reveals ontologically distinct host-pathogen interactions. Cell Rep.

[29] Gengenbacher M, Kaufmann SHE (2012). Mycobacterium tuberculosis: success through dormancy. FEMS Microbiol Rev.

[30] Nai A (2021). NCOA4-mediated ferritinophagy in macrophages is crucial to sustain erythropoiesis in mice. Haematologica.

[31] Santana-Codina N, Mancias JD (2018). The role of NCOA4-mediated ferritinophagy in health and disease. Pharmaceuticals (Basel).

[32] Ryu MS (2018). Ferritin iron regulators, PCBP1 and NCOA4, respond to cellular iron status in developing red cells. Blood Cells Mol Dis.

[33] Oke V, Wahren-Herlenius M (2012). The immunobiology of Ro52 (TRIM21) in autoimmunity: a critical review. J Autoimmun.

[34] Lee AYS (2021). Anti-Ro60 and anti-Ro52/TRIM21: two distinct autoantibodies in systemic autoimmune diseases. J Autoimmun.

[35] Yoshida M (2019). Involvement of cigarette smoke-induced epithelial cell ferroptosis in COPD pathogenesis. Nat Commun.

[36] Zhang Z (2018). Activation of ferritinophagy is required for the RNA-binding protein ELAVL1/HuR to regulate ferroptosis in hepatic stellate cells. Autophagy.

[37] Dixon SJ (2012). Ferroptosis: an iron-dependent form of nonapoptotic cell death. Cell.

[38] Minchella PA (2015). Complex anemia in tuberculosis: the need to consider causes and timing when designing interventions. Clin Infect Dis.

[39] van Lettow M (2005). Low plasma selenium concentrations, high plasma human immunodeficiency virus load and high interleukin-6 concentrations are risk factors associated with anemia in adults presenting with pulmonary tuberculosis in Zomba district, Malawi. Eur J Clin Nutr.

[40] Gangaidzo IT (2001). Association of pulmonary tuberculosis with increased dietary iron. J Infect Dis.

[41] Gordeuk VR (2002). African iron overload. Semin Hematol.

[42] Gordeuk VR (1996). Associations of iron overload in Africa with hepatocellular carcinoma and tuberculosis: Strachan’s 1929 thesis revisited. Blood.

[43] Isanaka S (2012). Iron status predicts treatment failure and mortality in tuberculosis patients: a prospective cohort study from Dar es Salaam, Tanzania. PLoS One.

[44] Isanaka S (2012). Iron deficiency and anemia predict mortality in patients with tuberculosis. J Nutr.

[45] Agoro R, Mura C (2019). Iron supplementation therapy, a friend and foe of mycobacterial infections?. Pharmaceuticals (Basel).

[46] Schaible UE (2002). Correction of the iron overload defect in beta-2-microglobulin knockout mice by lactoferrin abolishes their increased susceptibility to tuberculosis. J Exp Med.

[47] Kolloli A (2019). Effect of iron supplementation on the outcome of non-progressive pulmonary *Mycobacterium tuberculosis* infection. J Clin Med.

[48] Dai Y (2018). Empirical treatment with non-anti-tuberculosis antibiotics decreased microbiological detection in cervical tuberculous lymphadenitis. Diagn Microbiol Infect Dis.

[49] Dai Y (2020). Autoantibody-mediated erythrophagocytosis increases tuberculosis susceptibility in HIV patients. mBio.

[50] Cai Y (2021). Host immunity increases *Mycobacterium tuberculosis* reliance on cytochrome *bd* oxidase. PLoS Pathog.

[51] Gao P (2020). Peroxisome proliferator-activated receptor gamma (PPARγ) activation and metabolism disturbance induced by bisphenol A and its replacement analog bisphenol S using in vitro macrophages and in vivo mouse models. Environ Int.

[52] Zhu C (2019). Histone deacetylase inhibitors impair the host immune response against *Mycobacterium tuberculosis* infection. Tuberculosis (edinb).

[53] Mishra BB (2017). Nitric oxide prevents a pathogen-permissive granulocytic inflammation during tuberculosis. Nat Microbiol.

