# Supplementary material for: Mycobacterium tuberculosis hijacks host TRIM21- and NCOA4-dependent ferritinophagy to enhance intracellular growth
Source: J Clin Invest. 2023 Apr 17;133(8):e159941. doi: 10.1172/JCI159941 (PMC10104892; doi:10.1172/JCI159941)
Supplement: Supplemental data [file jci-133-159941-s127.pdf]

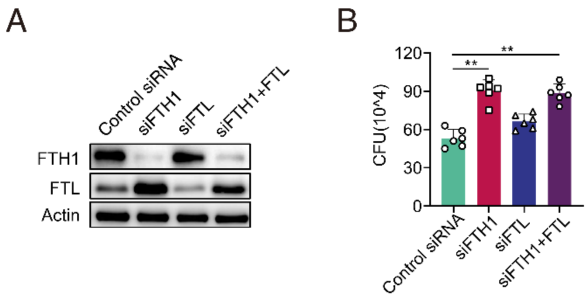

Supplemental Figure 1. FTL knockdown has no effect on intracellular CFUs of *M. tuberculosis*. (**A** and **B**) Immunoblot analysis of FTH1 and FTL (**A**) and intracellular H37Ra CFU levels (**B**) in macrophages transfected with FTH1 and/or FTL siRNA. Data are presented as means ± SD, ***$P < 0.001$, ****$P < 0.0001$, by one-way ANOVA with Tukey's post hoc test (**B**).

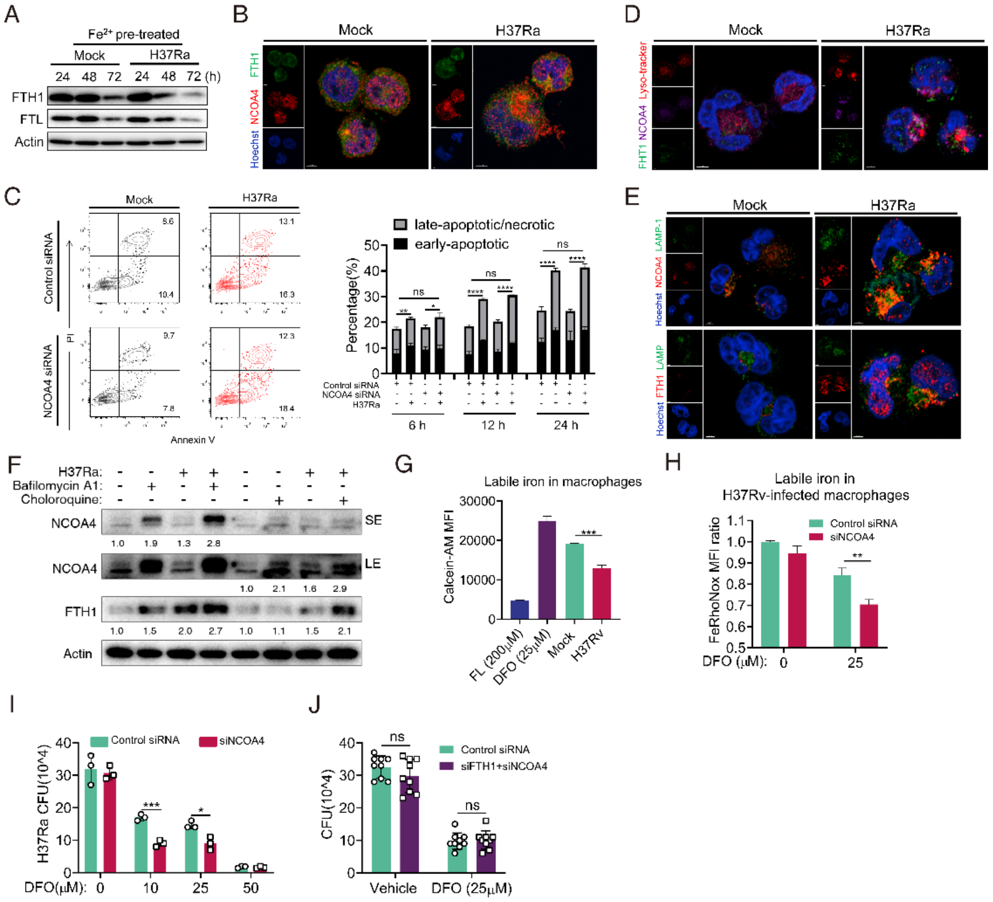

Supplemental Figure 2. Ferritinophagy enhances iron bioavailability and intracellular *M. tuberculosis* growth. (**A**) Immunoblot analysis of FTH1 and FTL at various time points (24, 48, and 72 h) in ferrous lactate (20 μM)-pretreated THP-1-derived macrophages. (**B**) Representative images of H37Ra-infected macrophages stained with NCOA4 and FTH1 (scale bars, 4 μm). (**C**) THP-1-derived macrophages were transfected with NCOA4 siRNA (50 nM). Scrambled siRNA was used as a negative control. Cell death was determined using an annexin V/propidium iodide (PI) kit following H37Ra infection (MOI = 10:1) for 6, 12, and 24 h by flow cytometry. (**D** and **E**) Representative images of H37Ra-infected macrophages stained with FTH1, NCOA4, and Lyso-tracker (scale bars, 4 μm). (**F**) Immunoblot analysis of NCOA4 and FTH1 in macrophages infected with H37Ra (MOI = 10) for 24 h, followed by bafilomycin A1 (100 nM) and chloroquine (25 μM) treatment for a further 3 h. (**G**) Detection of free iron by Calcein-AM in macrophages infected with H37Ra (MOI = 10) for 72 h or treated with controls including ferrous lactate (200 μM) and iron chelation, DFO (25 μM). (**H**) Detection of free iron by FeRhoNox in NCOA4-knockdown macrophages in the presence or absence of 25 μM DFO after H37Rv infection for 72 h. (**I**) Detection of intracellular H37Ra CFU levels in NCOA4-knockdown macrophages in the presence or absence of various concentrations of DFO (10, 25, and 50 μM) after infection for 72 h. (**J**) Detection of intracellular H37Rv CFU levels in NCOA4- and FTH1-knockdown macrophages in the presence or absence of DFO (25 μM) after infection for 72 h. Data are presented as means ± SD, **P* < 0.05, ***P* < 0.01, ****P* < 0.001, *****P* < 0.0001, by one-way ANOVA with Tukey's post hoc test (**G**) or Student's two-tailed unpaired t-test (**C, H-J**).

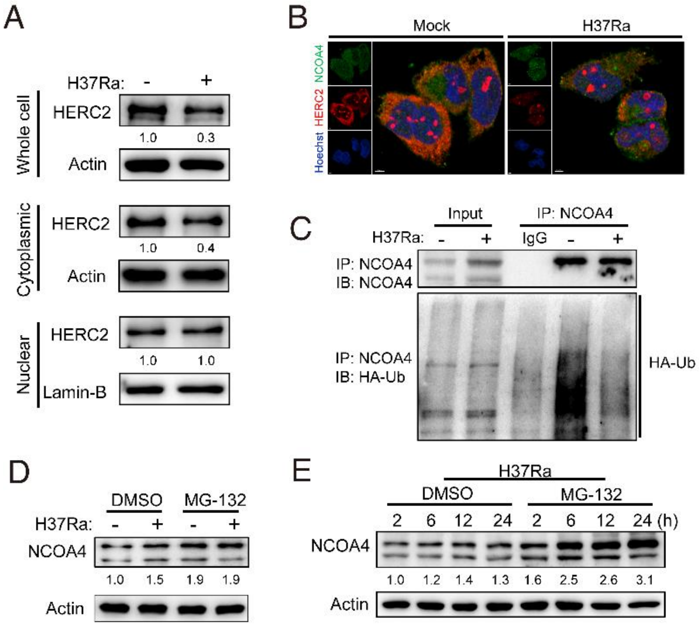

Supplemental Figure 3. *M. tuberculosis*-induced NCOA4 is regulated by HERC2-mediated proteolysis. (A) Immunoblot analysis of HERC2 in the cytoplasmic and nuclear fractions of control and H37Ra-infected macrophages. (**B**) Representative images of H37Ra-infected macrophages stained with HERC2 and NCOA4 (scale bars, 4 μm). (**C**) Immunoblot analysis and immunoprecipitation of NCOA4 and HA-Ub in macrophages transfected with HA-Ub plasmid followed by H37Ra (MOI = 10) infection for 24 h. (**D** and **E**) Immunoblot analysis of NCOA4 in macrophages infected with H37Ra (MOI = 10) for 6, 12, and 24 h in the presence or absence of MG-132 (2 μM) (**E**) or in macrophages infected with or without H37Ra (MOI = 10) for 24 h, followed by MG-132 (10 μM) treatment for a further 3 h (**D**).

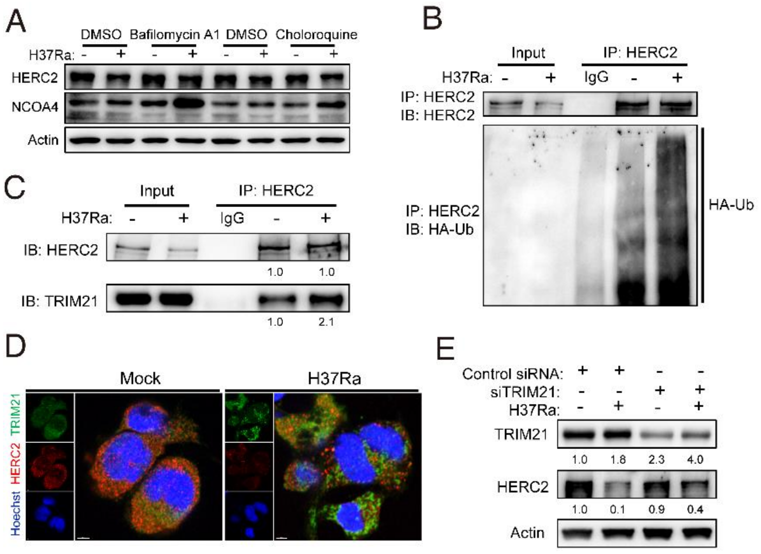

Supplemental Figure 4. *M. tuberculosis*-induced proteasomal degradation of HERC2 depends on TRIM21. (**A**) Immunoblot analysis of HERC2 and NCOA4 in THP-1-derived macrophages infected with H37Ra (MOI = 10) for 24 h, followed by bafilomycin A1 (100 nM) or chloroquine (25 μM) treatment for a further 3 h. (**B**) Immunoblot analysis and immunoprecipitation of HERC2 and HA-Ub in macrophages transfected with HA-Ub plasmid, followed by H37Ra (MOI = 10) infection for 24 h. (**C**) Immunoblot analysis and immunoprecipitation of HERC2 and TRIM21 in macrophages infected with H37Ra (MOI = 10) for 24 h. (**D**) Representative images of H37Ra-infected macrophages stained with HERC2 and TRIM21 (scale bars, 4 μm). (**E**) Immunoblot analysis of HERC2 and TRIM21 in macrophages transfected with TRIM21 siRNA and then infected with H37Ra (MOI = 10) for 24 h.

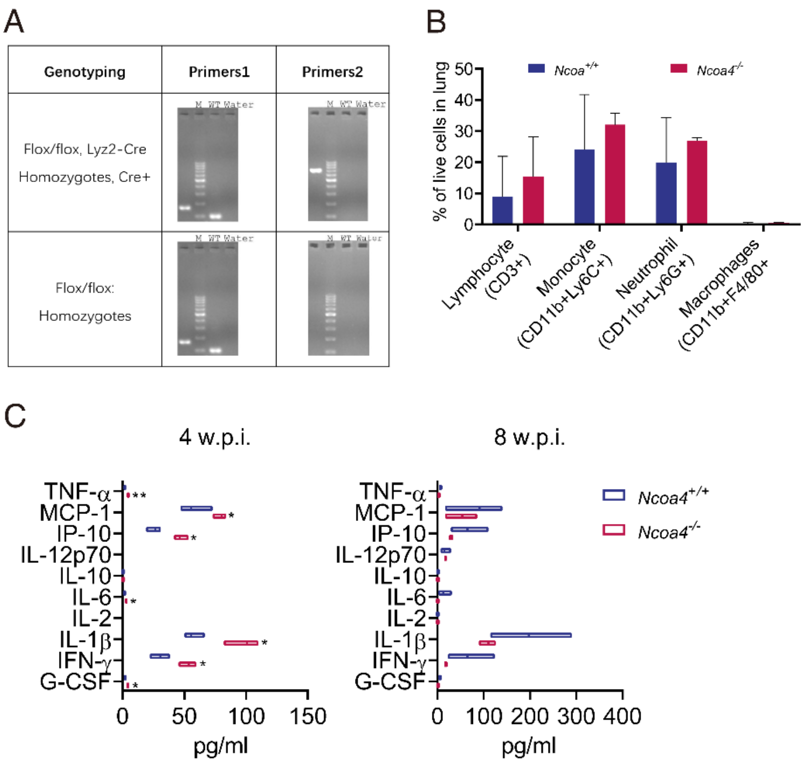

Supplemental Figure 5. NCOA4 deficiency in myeloid cells increases host inflammation response but does not affect lymphocyte, monocyte/macrophage, or neutrophil recruitment in response to *M. tuberculosis* infection. (**A**) PCR amplification showing the presence of the wild-type *Ncoa4* allele at 350 bp (*Ncoa4*$^{+/+}$) and the disrupted *Ncoa4* allele at 700 bp (*Ncoa4*$^{-/-}$). (**B**) The percentages of lymphocytes ($CD3^{+}$), monocytes ($Ly6C^{+}CD11b^{+}$), macrophages ($CD11b^{+}F4/80^{+}$), and neutrophils ($CD11b^{+}Ly6G^{+}$) in lungs from *Ncoa4*$^{+/+}$ and *Ncoa4*$^{-/-}$ mice infected with H37Rv were determined by flow cytometry. (**C**) Inflammation cytokines in the lung homogenates 4- and 8-weeks post-infection (w.p.i.) were detected by Luminex. Data are presented as means ± SD, *$P < 0.05$, by Student's two-tailed unpaired t-test (**B** and **C**).

Supplementary Table 1. Demographic characteristics of the study populations.

Cohort	No.	Age	Sex	Bacteriologically confirmed [a]	Histopathology [b]	Duration of anti-TB treatment before sample collection (months)
**Therapeutic resection**	T1	48	Male	Yes	+	2
	T2	35	Male	N/A	+	15
	T3	37	Male	Yes	+	6
	T4	22	Male	Yes	+	12
	T5	41	Female	Yes	+	13
	T6	63	Male	Yes	+	2
	T7	33	Male	Yes	+	<1
	T8	46	Male	Yes	+	6
	T9	53	Male	Yes	+	2
**Diagnostic biopsy**	D1	56	Male	Yes	+	None
	D2	45	Male	N/A	+	None
	D3	53	Male	N/A	+	None
	D4	42	Male	Yes	+	None
	D5	39	Male	N/A	+	None
	D6	40	Male	N/A	+	None

[a] Bacteriologically confirmed by acid-fast bacillus, Mtb culture, and nucleic acid amplification testing.

[b] Histopathology confirmed by the presence of typical caseous granuloma and multiple Langhans giant cells.

N/A: not applicable.

Supplementary Table 2. Potential E3 ligases for HERC2 identified by mass spectrometry analysis

Protein	Score	Mass	Matches	Sequences	emPAI
**TRIM21**	445	55162	35 (21)	15 (11)	0.94
**TRIM25**	53	72581	5 (3)	5 (3)	0.13
**ISG15**	39	17933	2 (1)	2 (1)	0.18
**UNKL**	33	75435	5 (1)	2 (1)	0.04
